# Anomalous behavior recognition of underwater creatures using lite 3D full-convolution network

**DOI:** 10.1038/s41598-023-47128-2

**Published:** 2023-11-16

**Authors:** Jung-Hua Wang, Te-Hua Hsu, Yi-Chung Lai, Yan-Tsung Peng, Zhen-Yao Chen, Ying-Ren Lin, Chang-Wen Huang, Chung-Ping Chiang

**Affiliations:** 1 Deptartment of Electrical Engineering, National Taiwan Ocean University, Keelung City, 20224 Taiwan; 2 AI Research Center, National Taiwan Ocean University, Keelung City, 20224 Taiwan; 3 Department of Aquaculture, National Taiwan Ocean University, Keelung City, 20224 Taiwan; 4 Center of Excellence for the Oceans, National Taiwan Ocean University, Keelung City, 20224 Taiwan; 5 Deptartment of Computer Science, National Chengchi University, Taipei, Taiwan

**Keywords:** Marine biology, Climate-change ecology, Behavioural ecology

## Abstract

Global warming and pollution could lead to the destruction of marine habitats and loss of species. The anomalous behavior of underwater creatures can be used as a biometer for assessing the health status of our ocean. Advances in behavior recognition have been driven by the active application of deep learning methods, yet many of them render superior accuracy at the cost of high computational complexity and slow inference. This paper presents a real-time anomalous behavior recognition approach that incorporates a lightweight deep learning model (Lite3D), object detection, and multitarget tracking. Lite3D is characterized in threefold: (1) image frames contain only regions of interest (ROI) generated by an object detector; (2) no fully connected layers are needed, the prediction head itself is a flatten layer of 1 × ${\mathcal{l}}$ @ 1× 1, ${\mathcal{l}}$= number of categories; (3) all the convolution kernels are 3D, except the first layer degenerated to 2D. Through the tracking, a sequence of ROI-only frames is subjected to 3D convolutions for stacked feature extraction. Compared to other 3D models, Lite3D is 50 times smaller in size and 57 times lighter in terms of trainable parameters and can achieve 99% of F1-score. Lite3D is ideal for mounting on ROV or AUV to perform real-time edge computing.

## Introduction

Global warming and extreme climate change are having a profound impact on our oceans and marine life. Their effects could lead to the destruction of marine habitats and loss of species. Exposure to environmental change and pollution can change the behavior of marine animals, e.g. exposure to elevated CO2 and reduced seawater pH can alter the behavior of reef fish and hermit crabs. In addition to climate change, other man-made factors such as low-frequency noise generated by giant wind turbines could adversely affect marine ecology. To tackle, the 14th goal of United Nations’ Sustainable Development Goals (SDGs)^1^ is aimed at the conservation and sustainable use of marine ecosystems. One key SDG is the predictable ocean, which has been launched^2^ to mobilize the ocean community to focus technological developments and research in oceanography on vital issues of protection and sustainable use of the ocean.

Since 2020, the coast of Taiwan has experienced severe coral bleaching, and about 30% were dead. A UN report^3^ predicted that Taiwan will become one of the most seriously bleached regions in the world, and it will also be the region with the earliest bleaching and the highest loss rate in East Asia. In response to the 14th SDGs, the authors of this paper along with scholars from four prestigious universities in Taiwan formed a cross-disciplinary team in 2021 to study the ecology of two most coral-rich areas, Kenting and Gonglia located in southern and northeast Taiwan, respectively. This government-funded research aims to develop AI models specifically for monitoring marine life and the environment. The models will be trained with various data sources collected using underwater cameras, sonar, and hydrophones that convert underwater sound waves to electrical signals. To name a few, imagery and acoustic data of selected marine life are simultaneously collected and their correlations analyzed; video data for training AI models to monitor the bleaching the health status of corals and the species and quantity of habituating organisms; image data for training AI models to identify the health status of Macroalgae, which is known capable of absorbing carbon from the atmosphere animated, etc.

The anomalous behavior of underwater creatures is an important indicator of an ecological system under a changing and even deteriorating process and can be used as a health meter for the status of oceans. For example, any weird or abnormal behaviors could indicate the coral reefs have suffered serious problems, enabling timely responses and necessary measures to be taken. Besides, in the aspect of marine life rehabilitation, an efficient monitoring system installed in the rehabilitation area should allow us to grasp any subtle changes in the health of creatures, as well as to timely reflect if their restoration progress is on the right or wrong track. Therefore, it is desired to have the capability of behavior recognition in conjunction with IoT-based environment parameters and the living states of macroalgae, to ensure more accurate and efficient ecological research on both coral reefs and oceans. The present paper reports our latest research results regarding the anomalous behavior recognition of marine life. The rationale of this work is rooted in the observation that, under the effect of global warming and climate change, one can expect that more stress-induced anomalous behaviors of life below water can be captured as imagery data. The long-term collection of such imagery data can be used to train AI models for detecting anomalous behaviors of marine life and thus assessing the ecological status of coral reefs.

Recent work and literature regarding marine life behavior are reviewed as follows. In the field of marine swarm behavior research, Herbert-Read et al.^4^ found that sailfish will break up sardine swarms and besiege smaller groups, driving them to the surface and attacking in turns. Later they found that when weak creatures were attacked by hunters, there would be protean escape behaviors, which came from the pressure of survival. Following that, noise disturbance experiments^5^ were conducted on bass and found that additional noise disrupts the ability of individuals to coordinate their actions with each other, which may in turn disrupt the collective dynamics of the fish school. Spampinato^6^ proposed combining the Gaussian mixture model and moving average to detect fish, and using a clustering algorithm to estimate movement trajectory. Later, that method was combined with event detection to analyze fish behavior under the effect of typhoons^7^. Although it can track a fish trajectory for behavior identification, the trajectory only retains the coordinates of the fish in each frame; the target object within the image frames and its posture features changes are literally neglected, which could greatly reduce the accuracy of behavior recognition.

All the aforesaid work relies on traditional algorithms, with no AI elements involved. In contrast to the recognition of marine life behavior recognition, deep learning (a subfield of AI) has long been applied to human action recognition. To name a few, the 3D convolutional neural networks (3DCNN)^8^ has one hardwired layer, three 3D convolution layers, two subsampling layers, and one fully connected layer. Instead of random initialization, the hardwired layer in 3DCNN is manually prepared using information including the optical flows between two successive frames. Using RGB video and optical flow features, an approach called Two-Stream Convolutional Networks^9^ was proposed to improve the accuracy of behavior recognition. C3D^10^ has 8 convolution, 5 max-pooling, and 2 fully connected layers, followed by a softmax output layer. All 3D convolution kernels are 3 × 3 × 3 with a stride of 1 in both spatial and temporal dimensions. LRCN^11^ combines CNN and LSTM (long short-term memory) to learn action recognition. Despite the inclusion of temporal information, many existing deep learning models render superior accuracy at the cost of high computational complexity and slow inference speed.

Methods of aforesaid AI-based studies^8–11^ all aimed at terrestrial action recognition, such as diving, bike riding, fall-off, etc., and they all treated the task as a classification problem using a sequence of images as an input entity. Since they are only designed for semantically classifying a collective action such as “fish is swimming”, it is not possible for these methods to detect anomalous behavior of any individual object within an image frame. Namely, these methods cannot provide information on the position, size, category, and orientation of multiple target objects. We conjecture that these features if properly processed into a time series, can form useful training data for learning recognition of anomalous behavior. To this end, the task of object detection can play a role in giving the 2-D location of multiple objects in the image, as well as the prediction of their labels. In fact, image classification and object detection are two core techniques in computer vision, their fundamental difference must be understood in order to understand our work.

**Figure 1:**
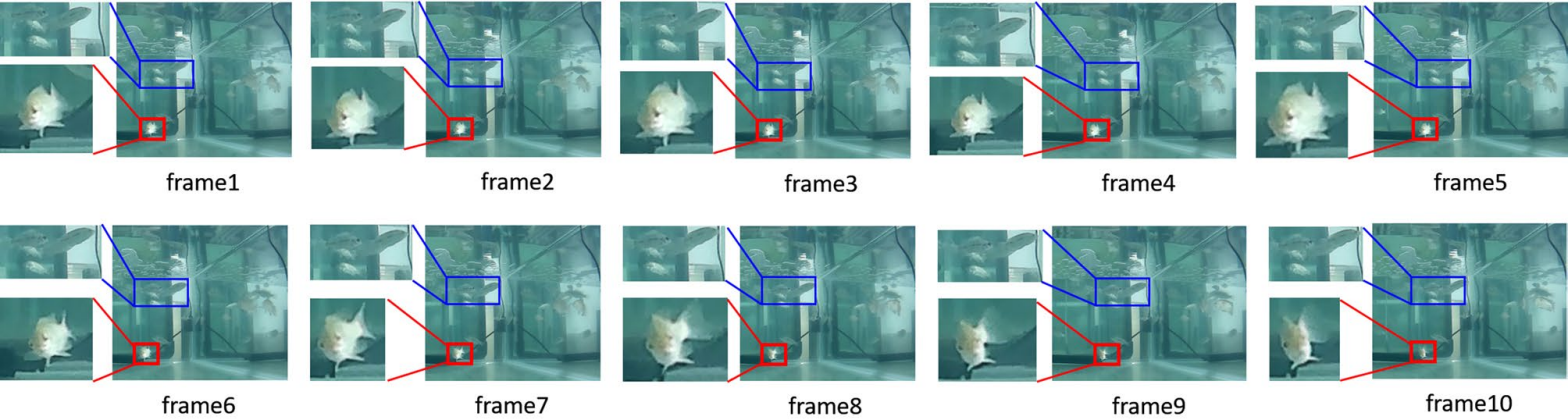
The posture sequence of a swimming fish, starting from 5th frame the fish body noticeably inclined to its right side, showing an anomalous behavior of *cartwheeling*.

Figure 1 shows a sequence of images used herein to contrast the difference, especially in the applicability to the field of marine life, between image classification and object detection. For clarity, red and blue bounding boxes detected via a trained object detector are zoomed. A time series of such boxes form a recognizable action called *cartwheeling*, which is an anomalous behavior commonly exhibited by illed tilapia. However, if we were using an image classifier rather than an object detector, each image frame would only be classified as a normal scene containing three fishes swimming therein, and the anomalous behavior of an individual fish would not be caught. This is because, compared to the entire image plane, an ill individual often takes up only a small portion of information insufficient to be correctly detected by the image classifier. For breeding and aquaculture experts, any anomalous behaviors such as the *cartwheeling* of individual tilapia should receive immediate attention. During the breeding process, it is desired to identify any individual fish exhibiting anomalous behavior that is caused by stress conditions set according to a specific experimental design. Because some brood fish are rare and very precious, sometimes early termination of experiments is necessary. Likewise, in the aquaculture industry, early detection of ill fish caused by bacteria or viruses is important because other fish in the same pool or tank could be rapidly infected.

## Motivation

**Figure 2:**
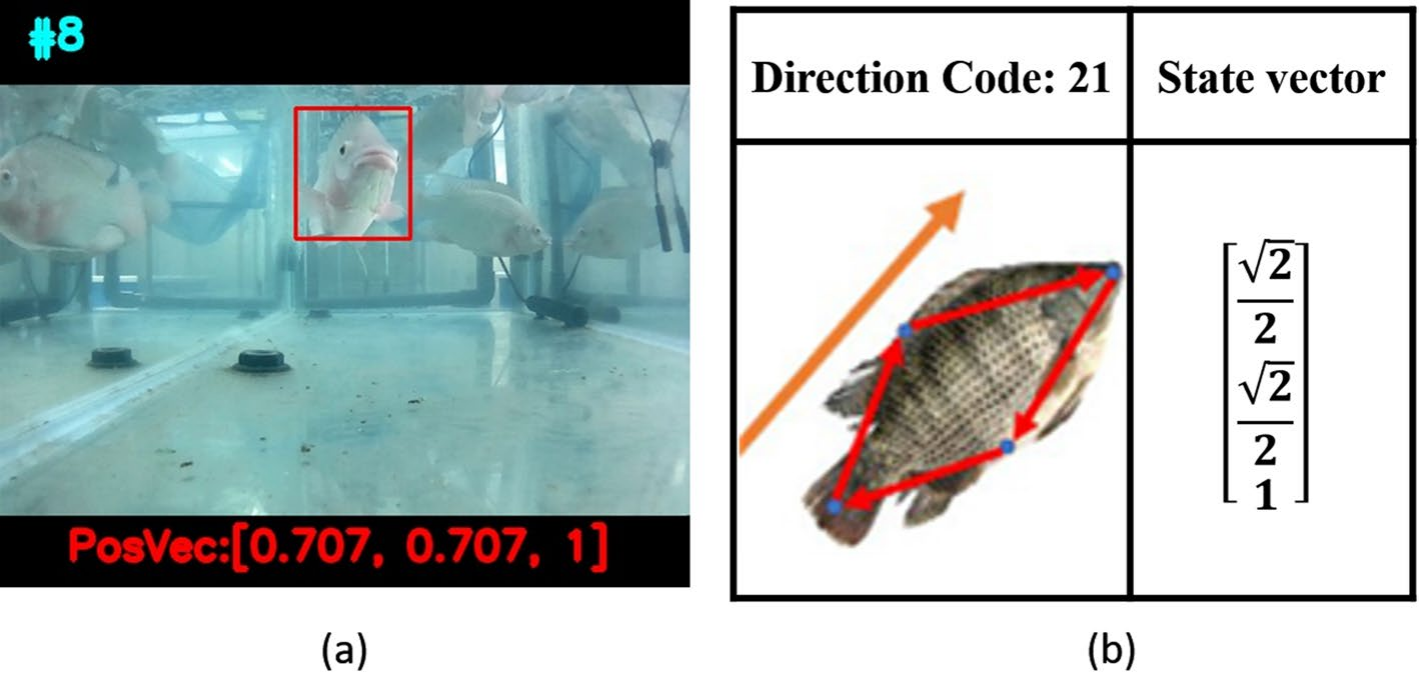
(**a**) A target fish swimming outwardly; (**b**) the erroneous state vector defined in DCG-DTW^12^.

To our best knowledge, DCG-DTW^12^ is the first attempt to apply object detection to the recognition of the individual behavior of fish, it utilized the property that the relative position of the fish's body parts remains unchanged to identify anomalous behaviors. A tracked fish will be represented by a sequence of encoded 3-D state vectors, with each vector representing the posture of the fish detected by an object detector. The vector defines the swimming direction as well as the positional relationship between key parts of fish: head, tail, fin, and dorsal fin. For example, in Fig. 2b a fish swimming northwest is encoded as $[\surd 2/2,\surd 2/{2,1}]$. Finally, through Dynamic Time Warping (DTW)^13^, a matching score between a template set and a state-vector sequence is calculated to determine if the fish tracked is abnormal. Though effective and simple, DCG-DTW suffers a problem when the fish is swimming at certain postures, such as heading toward or away from the reader. Figure 2a shows a fish swimming toward the reader, the state vector will be incorrectly encoded into the same vector as that in Fig. 2b. This is due to the fact that in DCG-DTW fish postures are defined in a 2D spatial plane, with the third coordinate of the 3D state vector only serving as the facing side indicator of the fish’s right eye. Thus, for the fish in Fig. 2a, though the dorsal fin is not apparent, we can still see that it is slightly on the left side of the fish head. In DCG-DTW, such posture will be mistakenly encoded. We also found that DCG-DTW may not only misjudge postures of the *cartwheeling* behavior, but the behavior of *side_swim* (swimming with one side of the fish body facing down), as some body parts other than fins in these postures cannot be successfully detected. Another limitation of DCG-DTW is its unsuitability for streamlined fish such as cobia because its dorsal fin is not easy to detect. Finally, the matching score calculation of DTW requires the sorting operation. If the total number of behavior classes (i.e. template size) increases, then the total computation time might easily grow to an extent that makes real-time edge computing applications virtually infeasible. In light of these observations, a new AI model is needed to directly learn spatiotemporal sequences of fish, so as to achieve accurate and real-time recognition of abnormal behavior.

To date, there are rather few reports of AI-based behavior recognition of marine life. Based on pre-extracted features of RGB and optical flow, Wang^14^ trains the C3D model and obtains fairly good behavior classification results. The shortcoming of C3D is that the input features need to be prepared manually prior to the deep learning, i.e., they are not automatically extracted. Thus, C3D cannot be trained directly with raw image data. Unlike the image classifier trained with optical-flow features^14^, Li et al.^15^ proposed an alternative solution based on the famous object detector YOLOv5. They added bidirectional feature pyramid network, coordinate attention block, and spatial pyramid pooling to YOLOv5, which was named BCS-YOLOv5. The method is characterized by using (mosaic) image fusion and modifying YOLOv5 to capture pose information of fish. In particular, it relies on explicitly defining four different behavior classes (normal, disease, hypoxia, and pH) for each object in order to perform anomaly detection of single fish in the input image. In contrast, our work is aimed to utilizing the results provided by an object detector to track a sequence of fish images, followed by classifying the input sequence as normal or not. That is, the main task of this paper is to develop a novel 3D classifier for recognizing the behavior of fish. Unlike 3DCNN and C3D of which training relies on not only raw imagery data but also images-derived features such as optical flow and gradients, the proposed Lite3D is built on a 3D full-convolution neural network which automatically extracts features, and can be combined with any state-of-the-art object detector and tracking algorithm to perform real-time recognition. Here, the term “full-convolution” is used to indicate no use of fully connected layers in the network architecture. Each fish is first detected as an ROI and tracked for certain image frames, and then the resulting sequence of ROIs extracted from the input video is fed to a cut-paste-warp scheme to produce a sequence of ROI-only frames for training the Lite3D to learn behavior recognition.

Lite3D is a lightweight model ideal for real-time detection of anomalous behaviors. Compared to its counterparts, Lite3D not only has the smallest model size and least number of trainable parameters but also is the fastest in execution speed. The main contributions of this work are *threefold*: (1) realize a novel approach that incorporates object detection, automatic feature extraction/classification, and tracking to perform real-time fish behavior recognition, without needing manual-derived features as well the error-prone vector encoding and time-consuming template matching; (2) present a lightweight 3D full convolution network, without the use of fully connected network at the prediction head; (3) devise a *cut-paste-warp* scheme to produce ROI-only sequential frames for training Lite3D effectively.

## Results

The experiment setup is listed as follows. Hardware: CPU/i7-8700 with one NVIDIA GPU/RTX-2080 and RAM/32G. Software: Windows 10, python 3.6.9, Cuda 10.2, cuDNN 7.6.5. Object detector uses YOLOv4-tiny under Tensorflow 1.14.0. Key hyperparameters of batch size, epoch, learning rate, and optimizer are set to 32, 50, 0.001, and Adam, respectively. Performance comparison is conducted using a private dataset prepared through the step of data preparation (elaborated later). This private dataset was labeled by aquaculture experts and used for the training and testing processes (see Supplementary Table S1, where we have 5862 ROI-only sequences, each having a length of ten frames) for cobia and 5596 sequences for tilapia. We see some categories have much less data than others, e.g. the “anomalous” in cobia and the “side_swim” in tilapia. As well known, the problem of the imbalanced dataset can be alleviated through data augmentation, under-sampling the majority class, using F1-score, etc. Note that the experiment is performed in compliance with performed in compliance with Taiwanese animal protection act, and approved by the Institutional Animal Care and Use Committee (IACUC-09053). All the authors followed the ARRIVE guidelines.

Also, for a fair comparison, we adjusted the input data dimension of 3DCNN^8^ and C3D^10^ to be the same as that of Lite3D. Since our goal is to make Lite3D deployable on an edge computing device for real-time recognition, performance criteria should include precision, recall rate, F1-score, model size, the total number of trainable parameters, and finally, the execution time. Note that the original C3D architecture requires over 78 M trainable parameters, which is too fat for limited computing hardware. For comparison purposes, we implemented a lite version of C3D that is close to 3DCNN in terms of model size and parameters. After the network construction and training, the model size and the number of trainable parameters for 3DCNN, C3D, and Lite3D are calculated by Tensorflow, and they are 16.3 MB/4.27 M, 18.6 MB/4.88 M, and 349 KB/0.08 M, respectively. Specifically, in terms of model size, Lite3D is 50 times smaller than the other two models; and in terms of trainable parameters, Lite3D is 57 times lighter.

To compare the performance of the three models, we used a private dataset that contains samples of three behavior classes for cobia, and four for tilapia in Table 1. Regarding the loss function during the backpropagation training process^16^, both “cross entropy” and “focal loss”^17^ were tested. For the former, although all three models can achieve nearly 96% in average precision, they had only 92% precision for the “grinding” class of tilapia, because the data quantity thereof is relatively small (only 53 for training and 13 for testing, see Supplementary Table S1). To improve, we substitute the focal loss for the cross entropy, the precision for the “grinding” class of tilapia has increased to 100%, and the average precision of all three models increased from 96 to 98%.

**Table 1:** Recognition performance comparison.

Model	Precision (for individual behavior category)							Precision	Recall	F1-score
	Cobia			Tilapia						
	Normal	Lifeless	Anomalous	Normal	Grinding	Cartwheeling	Side_swim			
3DCNN	0.97	0.99	1.00	0.99	1.00	0.97	0.91	0.97	0.96	0.97
C3D	0.98	0.99	1.00	0.98	1.00	0.93	1.00	0.98	0.98	0.98
Lite3D	0.98	0.99	1.00	0.99	1.00	0.95	1.00	0.98	0.99	0.99

To help readers better understand the necessity as well as the effectiveness of using the cut-paste-warp scheme, we have also prepared a dataset without using cut-paste-warp for comparison purposes. Table 2 shows the training results of the three models using the focal loss. By comparing Tables 1 and 2, it is easy to see that all models suffer a significant performance degradation for the “grinding” class of tilapia. This is due to the close resemblance of the “grinding” class to the “cartwheeling” class in terms of moving poses. The most noticeable difference is that the “grinding” class always occurs at the bottom of the tank. Namely, anomalous behaviors occurring at the bottom of the image are likely predicted as the “grinding” class. This result justifies the use of ROI-only frames generated via the cut-paste-warp scheme. Finally, Table 3 provides a performance comparison between methods. Lite3D outperforms in terms of precision, recall, F1-score, FPS, and the number of free parameters. Note that, compared to 3DCNN, C3D, and Lite3D which all involve explicitly learning temporal information, BCS-YOLOv5 yields the lowest precision. This is because BCS-YOLOv5 is solely based on object detection, which inevitably gives more false-positive output predictions.

**Table 2:** Recognition performance comparison without using the cut-paste-warp scheme.

Model	Precision for individual behavior class							Precision	Recall	F1-score
	Cobia			Tilapia						
	Normal	Lifeless	Anomalous	Normal	Grinding	Cartwheeling	Side_swim			
3DCNN	0.96	0.99	1.00	0.99	0.92	0.94	0.91	0.96	0.98	0.97
C3D	0.96	0.99	0.89	1.00	0.81	1.00	0.83	0.93	0.92	0.92
Lite3D	0.93	0.98	1.00	0.99	0.86	0.99	1.00	0.97	0.90	0.93

**Table 3:** Single fish behavior detect performance comparison.

Method	Precision	Recall	F1-score	mAP50 (Detector)	FPS	Num. of parameters (Detector /Classifier)
Yolov4 + 3DCNN	0.97	0.96	0.97	0.78	38.59	27.6 M/ 4.2 M
Yolov4 + C3D	0.98	0.98	0.98	0.78	38.21	27.6 M/4.8 M
Yolov4 + Lite3D	0.98	0.99	0.99	0.78	38.87	27.6 M/0.08 M
Faster-RCNN + DCG-DTW	0.84	0.80	0.82	0.50	27.26	6.5 M/NA
Yolov4 + DCG -DTW	0.79	0.56	0.66	0.35	17.38	27.6 M/NA
EfficientDet-D1 + DCG -DTW	0.55	0.61	0.58	0.41	17.13	6.6 M/NA
BCS-YOLOv5	0.93	0.93	0.93	0.97	55	9 M/NA

**Figure 3:**
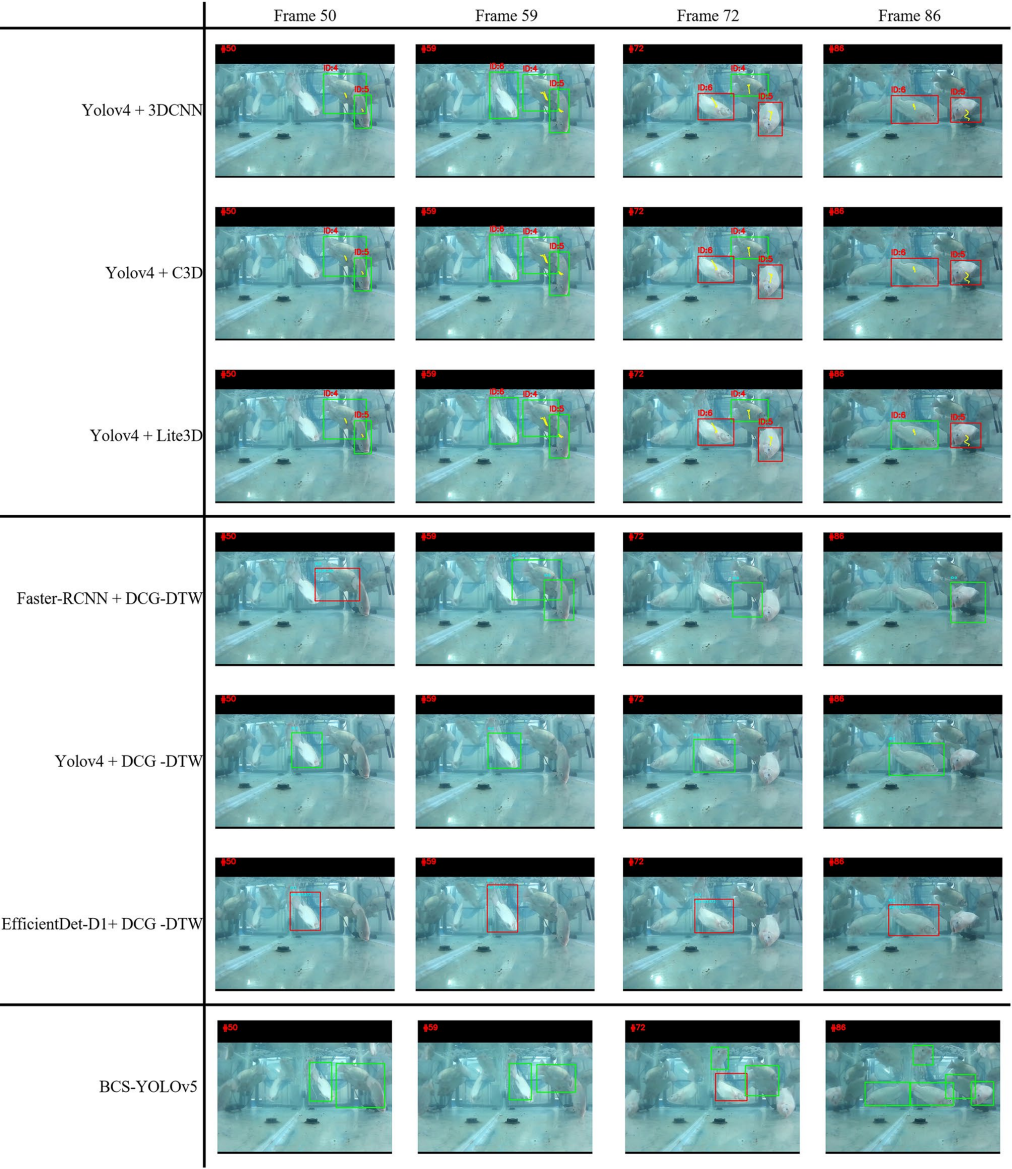
From top to bottom are the recognition comparisons of yolov4 + 3DCNN, yolov4 + C3D, yolov4 + Lite3D, Faster-RCNN + DCG-DTW, yolov4 + DCG-DTW, EfficientDet-D1 + DCG-DTW and BCS-YOLOv5**.**

Using a test video of four image frames containing multiple fish, Fig. 3 pictorially shows the recognition results of the three models trained with “focal loss”. Here, yellow lines represent the tracking traces, while green and red boxes indicate normal and anomalous behaviors detected, respectively. In the 72^nd^ frame, we see that ID_5 and ID_6 respectively exhibit “grinding” and “cartwheeling” for all three models. Using naked eyes, we can see that in frame 86, fish ID_6 already returned to a “normal” swimming posture, while fish ID_5 remained at “side_swim”. However, Fig. 3 shows that only Lite3D successfully detected the fish ID_6 returning to “normal” at frame 86 (indicated by the green box), justifying that our method indeed performs better over the other two models in differentiating between normal and anomalous behaviors of fish.

As shown in Table 4, the proposed lightweight model is 3 to 5 times faster than the object detector YOLOv4-tiny, and our recognition method can reach 98 fps on RTX2080. If run on Jetson Xavier™ NX, it can still achieve 39 fps, fully justifying its feasibility for realizing edge-computing applications on ROV or AUV.

**Table 4:** Execution time breakdown of the proposed method (in secs).

Device	Object detection	Tracking	Lite3D	Total time	FPS
Jetson Xavier™ NX (CPU NVIDIA Carmel Arm®v8.2, GPU NVIDIA Volta™)	0.01762	0.00222	0.00541	0.02525	39.60
PC (CPU Inteli7-8700, GPU RTX 2080 × 1)	0.00769	0.00091	0.00159	0.01020	98.02

## Discussion and conclusion

The total number of trainable weights used by Lite3D is only 1/57 that of 3DCNN and C3D. In terms of model size (i.e. memory space required to store the total number of network layers and artificial neurons), Lite3D is only 1/50 of 3DCNN and C3D. Such a tiny model is achieved at no expense of recognition performance, and it can be used with any state-of-the-art object detector and tracking algorithm to achieve 99% in F1-score.

Because warping is conducted on an ROI-pasted blank canvas and the result is used as input data to Lite3D, both spatial and temporal information is well preserved. Spatiotemporal info is extracted through plural 3D convolution layers. All convolution layers of Lite3D adopt random initialization, so no need to manually derive features of optical flow and gradients as the hardwired input layer in 3DCNN. In contrast, intermittent postures erroneously detected in DCG-DTW could result in poor accuracy. As shown in Fig. 3, where two or three fish were being detected and tracked during 50th–86th frames, if DCG-DTW were used, fish ID_5 at frame 72 would be falsely identified as though no changes in posture due to no parts of the fish were detected. Since pre-defined states are not necessary for Lite3D, this problem of mis-detected body parts is not an issue at all. For future work, the representational power of Lite3D may be further improved by invoking Squeeze-and-Excitation Networks^18^ to adaptively recalibrate channel-wise feature responses after layer ${C}_{1}$ (see “Methodology”). Also, the computational cost be further reduced by decomposing all the standard 3D convolutions into depth-wise and pointwise convolutions^19^. Finally, it should be worth trying to apply our method to terrestrial action recognition and compare its performance with those of methods^8–11^.

The presentation of anomalous behaviors in marine life often indicates symptoms of disease or signs of being under stress due to coldness, too salty water, etc. In light of this, our method can also be used to assist the breeding and aquaculture industry in providing an early warning of abnormal or emergency conditions that require timely measures to prevent significant losses. Also, it is fairly easy to integrate the software coding of our method with a cost-saving camera and IMU (inertial measurement unit) to produce affordable underwater monitoring devices. Having said that, we should be cautious that in practice, the performance of our method is inevitably influenced by the performance of the detector and tracking. Thus, it is very important to have a well-performing object detector and tracking algorithm in the presence of complex underwater conditions. For example, if the object detector lacks a sufficiently high sensitivity in detecting marine life (e.g., in facing a very complex underwater background), we may come into an interruption of tracking sequence, hence resulting in an inability to identify behaviors correctly. To this end, we will study the plausibility of merging the object detector, tracking, and Lite3D to strengthen the system robustness through the implementations of weigh-sharing as well as the end-to-end fusion^20^ in the aspect of architecture, rather than the purely spatial fusion using the input images^15^.

## Methodology

**Figure 4:**
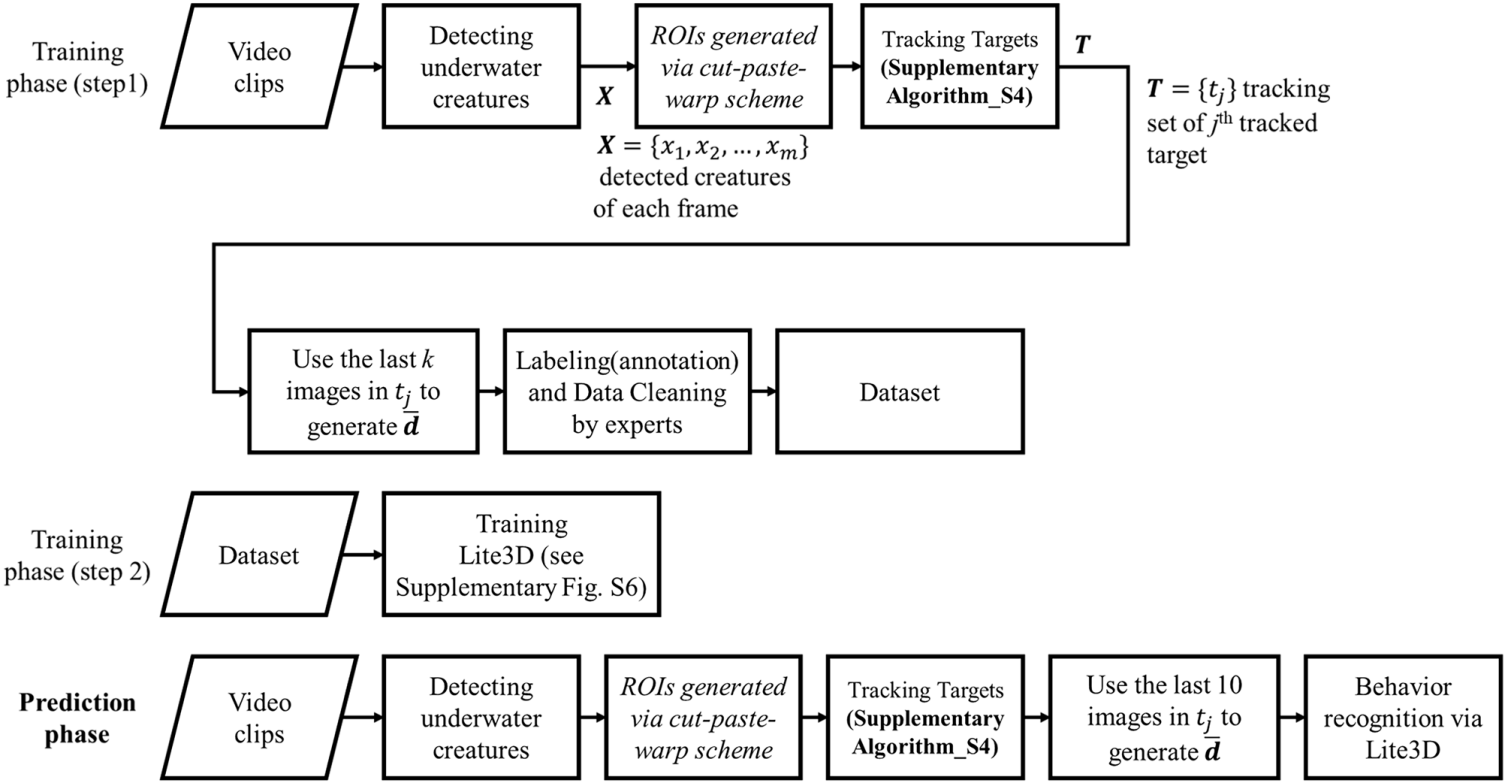
Flowchart of the proposed anomalous behavior recognition method.

The flowchart of the proposed method is shown in Fig. 4, it consists of two phases: Training and Prediction. The former further comprises two steps: data preparation and model training. In **Step_1**, an object detector is used to perform the detection of target creatures bounded by an ROI (Region of Interest) for each image frame. Each individual target will be tracked within the frames to generate a sequence of ROIs. Each ROI will be processed by the *cut-paste-warp scheme*, i.e., it will be pasted back to its corresponding position of the original input frame with the background completely blackened out. A training vector $\overline{{\varvec{d}} }$= (*d*_*k-9*_,* d*_*k-8*_,*…,d*_*k*_) can be obtained by extracting *k* images in a sequence of ROIs. Without loss of generality, set *k* = 10 and choose YOLOv4-tiny^21, 22^ as the object detector, and use cobia and tilapia as the demonstrating fish species. Then, a behavior category is assigned, by experts, to $\overline{{\varvec{d}} }$. Following that, experts are asked to screen, based on a majority voting scheme, those data with confusing annotations. In **Setp_2**, the cleaned data is divided into a training set and a test set, and Lite3D is built and trained using the training set. Finally, in the prediction phase, frames of video are streamed into the trained Lite3D for behavior recognition. First, target objects in successive frames are detected and tracked to obtain ROIs (i.e. any red or green bounding box in Fig. 3), thus generating a sequence $\overline{{\varvec{d}} }$ for each tracked target for the trained Lite3D to perform prediction. The method is elaborated as follows.

## Step_1 Data Preparation

### Annotation of underwater creature behaviors

Supplementary Fig. S3a shows a false detection of body parts by DCG-DTW^12^, where the tail of one fish (the smaller brown box) was erroneously identified as belonging to another fish (red box). This error is rooted in that the center point of the tail is located within the red bounding box. Also, as explained earlier, DCG-DTW is unable to define the two postures in Supplementary Fig. S3b and Supplementary Fig. S3c, namely, fish swimming toward and away from the reader. The proposed method can do without the aforesaid problems.

We have collected and annotated video data of various marine life including tilapia, cobia, crab, lobster, cuttlefish, echinus, and sea cucumber. However, to help readers understand our work, in this paper we only present results of tilapia and cobia. At this moment, there are seven categories defined by experts, three for cobia and seven for tilapia. Exemplar categories and their posture sequence are illustrated in Supplementary Fig. S4, where ${d}_{i}$ indicates a target fish detected at the *i*th frame. Supplementary Fig. S4a shows sequential movement of “normal” behavior. Supplementary Fig. S4b shows an anomalous behavior of side_swim, meaning the fish is swimming with one side of its body facing down, hence the name. Supplementary Fig. S4c shows a fish remaining still for a period of time at the pool bottom. Because cobia is migratory fish, if a cobia stays unmoved for a long time, then it deserves attention. This kind of behavior is annotated as “lifeless “. Supplementary Fig. S4d depicts a fish rubbing against the pool bottom, with the body being unbalanced or flipped. It is annotated as the “grinding” category in tilapia, and the “anomalous” category in cobia. If a cobia exhibits the grinding behavior, likely there are parasites on the skin; Supplementary Fig. S4e shows a fish exhibiting a toppling posture, it is annotated as “cartwheeling”.

### ROI-only frames generated via cut-paste-warp scheme

State-of-the-art deep neural networks often require fixed-size input images, and so does the proposed Lite3D. However, we cannot simply use the cutout ROIs (see Supplementary Fig. S5a) and warp them into the same size (Supplementary Fig. S5b) for training Lite3D, because doing so will cause the loss of *spatially varying* information that is essential to the behavior recognition. To see this, we first note that any edge detectors would produce ROIs of various sizes, as shown in Supplementary Fig. S5a. If one simply applies warping to ROIs to reshape them into the same size, then temporal information originally inferable from the consecutive input frames (i.e., changes in body size and orientation), is likely destroyed. This can be seen by comparing Supplementary Fig. S5a and Supplementary Fig. S5b, where the body-moving variations of the fish are lost in Supplementary Fig. S5b due to the warping. We solve this problem by preparing a black canvas having the size of the original image frame, then pasting the ROI to the canvas at the position where the ROI is originally located, and then warping the ROI-pasted canvas to the size required by the network in question. The result is shown in Supplementary Fig. S5c where moving variations of the fish are nicely kept. The performance comparison of using ROIs generated without applying the cut-paste-warp scheme (Supplementary Fig. S5b) and ROI-only frames generated by applying the cut-paste-warp scheme (Supplementary Fig. S5c) as test datasets are shown in Tables 1 and 2, respectively. Finally, data with confusing annotations will be removed through majority voting by aquaculture experts, resulting in 5,862 sequences for cobia and 5,596 sequences for tilapia(see Supplementary Fig. S7 for examples of the seven categories).

## Step 2 Model training

Without loss of generality, we explain the network design of Lite3D using the exemplar configuration Supplementary Fig. S6. Feature maps at different layers of Lite3D is shown in Supplementary Fig. S6a, and the architecture configuration is depicted in Supplementary Fig. S6b Lite3D has a backbone and a prediction head ${\text{C}}_{4}$. The network configuration of Lite3D is shown in Supplementary Fig. S6b, it has a backbone (green boxes together) and a prediction head (red box). The backbone, which acts as a spatiotemporal feature extractor, comprises three blocks, each having a convolutional layer ${\text{C}}_{\mathrm{i}}$ and a Max-pooling layer ${\text{S}}_{\mathrm{i}}$. The feature maps generated by ${\mathrm{C}}_{1}, {\mathrm{S}}_{1}, {\mathrm{C}}_{2}, {\mathrm{S}}_{2}, {\mathrm{C}}_{3}, {\mathrm{S}}_{3}$, and ${\mathrm{C}}_{4}$ are expressed in tensor format $k\times c@n\times n$, where *c* may represent the number of channels or it may refer to the number of kernels (and hence the number of feature maps). The integer $k$ is the number of $n\times n$ image frames in the input video, it may also refer to the number of feature maps generated by the previous layer. Though other integer values may also be used, here for illustration purposes we set *k* = 10, c = 3, *n* = 64 for the input layer in Supplementary Fig. S6. The key feature of this design is that we substitute convolutional layers for full-connection (FC) layers at the prediction head. Especially, the convolution kernel applied to the flatten layer has the size of $1\times {\mathcal{l}}@1\times 1$, ${\mathcal{l}}$=the number of categories, thus enabling the replacement of FC layers simply with a flatten layer. One merit of such a design is that it substantially reduces the number of trainable weights, as verified in section “Results”. In addition, our design allows $k$ to be calculated by the model architecture which is normally preconfigured by the network developer. The following discussions briefly describe how. First, we approach this problem by starting with a broader question: can the proper values of $(w, h, k)$ be determined if the kernel size of each convolutional layer and that of pooling layer are known? Take Supplementary Fig. S6a as an example, where Lite3D is designed to have four convolutional layers ${\mathrm{C}}_{1}$, ${\mathrm{C}}_{2}$, ${\mathrm{C}}_{3}$, ${\mathrm{C}}_{4}$ and three pooling layers ${\mathrm{S}}_{1}$, ${\mathrm{S}}_{2}$, ${\mathrm{S}}_{3}$. We assume the kernel size of 3D convolution at ${l}{th}$ is ${\mathrm{c}}_{{n}_{l}}\times {c}_{{n}_{l}}\times {c}_{{p}_{l}}$, the kernel size of pooling layer ${p}_{{n}_{l}}\times {p}_{{n}_{l}}\times {p}_{{p}_{l}}$, and the final size of feature map is $w^{\prime}\times h^{\prime}\times k^{\prime}$. Further, assume no zeros padding. The size parameters of input frame $h$ and $w$ are calculated as Eq. (1), and the depth *k* in the temporal direction is Eq. (2). The integer value of $w$ and $k$ can be given located in a range through Eq. (3) and Eq. (4).

1 $$w = h = c_{{n_{1} }} - 1 + p_{{n_{1} }} \left( {c_{{n_{2} }} - 1 + p_{{n_{2} }} \left( {c_{{n_{3} }} - 1 + p_{{n_{3} }} \left( {c_{{n_{4} }} - 1 + w^{{\prime }} } \right)} \right)} \right)$$

2 $$k = c_{{p_{1} }} - 1 + p_{{p_{1} }} \left( {c_{{p_{2} }} - 1 + p_{{p_{2} }} \left( {c_{{p_{3} }} - 1 + p_{{p_{3} }} \left( {c_{{p_{4} }} - 1 + k^{{\prime }} } \right)} \right)} \right)$$

3 $$c_{{n_{1} }} + p_{{n_{1} }} \left( {c_{{n_{2} }} - 1 + p_{{n_{2} }} \left( {c_{{n_{3} }} - 1 + p_{{n_{3} }} \left( {c_{{n_{4} }} - 2 + w^{{\prime }} } \right)} \right)} \right) \le w \le c_{{n_{1} }} - 1 + p_{{n_{1} }} \left( {c_{{n_{2} }} - 1 + p_{{n_{2} }} \left( {c_{{n_{3} }} - 1 + p_{{n_{3} }} \left( {c_{{n_{4} }} - 1 + w^{{\prime }} } \right)} \right)} \right)$$

4 $$c_{{p_{1} }} + p_{{p_{1} }} \left( {c_{{p_{2} }} - 1 + p_{{p_{2} }} \left( {c_{{p_{3} }} - 1 + p_{{p_{3} }} \left( {c_{{p_{4} }} - 2 + k^{{\prime }} } \right)} \right)} \right) \le k \le c_{{p_{1} }} - 1 + p_{{p_{1} }} \left( {c_{{p_{2} }} - 1 + p_{{p_{2} }} \left( {c_{{p_{3} }} - 1 + p_{{p_{3} }} \left( {c_{{p_{4} }} - 1 + k^{{\prime }} } \right)} \right)} \right)$$

Because the desired final size of feature map is ${w}^{\prime}\times {h}^{\prime}\times {k}^{\prime}=1\times 1\times 1,$ with ${\mathrm{c}}_{{n}_{1}}={\mathrm{c}}_{{n}_{2}}={\mathrm{c}}_{{n}_{3}}={\mathrm{c}}_{{n}_{4}}=3,{p}_{{n}_{1}}={p}_{{n}_{2}}=3,{p}_{{n}_{3}}=2$, using Eq. (3) yields $63\le w, h\le 80$. That explains why in Supplementary Fig. S6, the input frame size was set to 64. Likewise, with ${c}_{{p}_{1}}=1, {c}_{{p}_{2}}={c}_{{p}_{3}}={c}_{{p}_{4}}=3,{p}_{{p}_{1}}={p}_{{p}_{2}}=1,{p}_{{p}_{3}}=2$, using Eq. (4) gives us $9\le k\le 10$. Accordingly, in Supplementary Fig. S6, the depth *k* was set to 10. Clearly, given the equations of (1) through (4), if one is given the value of parameters of $h, w, k$ under the constraint of the value of ${w}^{\prime}, {h}^{\prime}, {k}^{\prime}$ all being one, the problem of finding out the ${\mathrm{c}}_{{n}_{l}},{c}_{{p}_{l}},{\mathrm{c}}_{{n}_{l}},{c}_{{p}_{l}}$ becomes solving four simultaneous equations. Unfortunately, the reverse is not always possible. That is, given a* k* value, no analytical solution exists yet. However, the techniques of NAS (network architecture search) can be used for searching plausible network configurations.

The rationale for replacing all FC layers is rooted in two observations^23^: (1) the convolutional layer learns better with the underlying information of the image data, thus enabling lower training loss with fewer trainable parameters (2) fewer trainable parameters make possible better resilience to overfitting and hence better generalization capability, especially in the case of few training data available. Using entry-wise independent Gaussian and CIFAR-10 for the test, they also showed that in dealing with simple classification or object detection tasks, *all-convolution* networks perform better than hybrid networks (i.e. CNN + FC). However, we note that all-convolution networks such as AConvNet^24^ are mainly aimed at tackling 2-D image classification tasks. In order to handle spatiotemporal data, 3D convolution models are capable of providing the required representation capability, yet they inevitably incur massive trainable parameters, making it difficult to be deployed in real-time edge computing applications.

In Supplementary Fig. S6b, the 3D kernel size of all convolution and max-pooling is expressed as $h\times w\times p$, where *p* represents the depth in the temporal direction. That is, $h\times w\times p$ means the $h\times w$ kernel being applied simultaneously to $p$ successive frames to generate an output feature map. The padding of all layers was set to “*valid*”, and the stride of (1, 1, 1). With *k* input frames, we will have $k-(p-1)$ output frames. In Supplementary Fig. S6b, all convolutional layers adopt $3\times 3\times 3$ kernel size, except the ${\mathrm{C}}_{1}$ layer, which is purposely degenerated to $3\times 3\times 1$. Namely, with $p =1$ in ${\mathrm{C}}_{1}$ and the input video clip is assumed to have 10 frames, each of which has three channels (RGB). Further, assuming there are 16 convolution kernels in ${\mathrm{C}}_{1}$, there will be 16 output channels for the input video. Because all the convolution kernels in ${\mathrm{C}}_{1}$ have a size of $3\times 3\times 1$, each time the convolution is applied on only one frame in the depth direction, no temporal information will be collected until the ${\mathrm{C}}_{2}$ layer. In short, the ${\mathrm{C}}_{1}$ layer is only responsible for extracting spatial features needed for the truly 3D convolutions in ${\mathrm{C}}_{2}$ through ${\mathrm{C}}_{4}$ layers to stack temporal information.

The output feature maps of the first block can be shown to be $10\times 16@21\times 21$, and frames, the total number of trainable parameters for the first block is calculated as $3\times 3\times 3\times 16=432$ added by 16 biases, which equals 448. At layer ${\mathrm{C}}_{2}$, 32 kernels of $3\times 3\times 3$ are used, with $p=3$, there will be 8 output frames. And because the kernel of the ${\mathrm{S}}_{2}$ layer is set to $3\times 3\times 1$, the output of the second block is a tensor of $8\times 32@7\times 7$ and 13,856 trainable parameters. Likewise, the output of the first block has the tensor expression of $3\times 64@3\times 3$ and 55,360 trainable parameters. Thus, the total number of trainable parameters is calculated as Eq. (5):

5 $$69{,}664+{1728}\times {\mathcal{l}}+{\mathcal{l}}$$

With ${\mathcal{l}}$=7, the total number of trainable weights in Supplementary Fig. S6 thus equals 0.08 M. The 3D convolution is prescribed in Eqs. (6) and (7), where ${I}_{j}^{l}\left(x, y, z\right)$ is the *j*th feature map in the *l*th layer; $f$ is the activation function, and ${V}_{j}^{l}(x, y, z)$ is the convolution result of the *j*th feature map in the *l*th layer; ${w}_{j, c}^{l}$ represents kernel weights connected between the *j*th feature map in the *l*th layer and the *c*th feature map of the previous layer.

6 $${I}_{j}^{l}\left(x, y, z\right)=f({V}_{j}^{l}(x, y, z))$$

7 $${V}_{j}^{l}\left(x, y, z\right)={b}_{j}^{l}+\sum_{c}\sum_{p=0}^{h-1} \sum_{q=0}^{w-1}\sum_{r=0}^{dp-1}{w}_{j, c}^{l}\left(p, q, r\right)*{I}_{c}^{l-1}(x-p, y-q, z-r)$$

Note the gradients for the kernel weights are computed using the famous backpropagation algorithm, except the same weights are shared across many connections of frames or feature maps at the previous layer, see (red, blue, and green) arrows in Supplementary Fig. S6c. The flatten layer uses focal loss^17^ of Eq. (8), where $0<{\alpha }_{i}$≤1 represents the weight of the ${i}{th}$ category loss, which is responsible for solving the problem of the imbalanced number of categories; and ${\left(1-A({x}_{i})\right)}^{\gamma }$ represents the loss adjustment factor, which is responsible for solving the problem of learning difficult data. By adjusting $\gamma$ to reduce the loss corresponding to easy-to-learn data, the model can focus on learning complex data. Assuming ${\alpha }_{i}=1$, Supplementary Fig. S8 shows the effect of using different values of γ on the model loss. Regardless of the value of ${\alpha }_{i},$ a larger $\gamma$ tends to make the model saturate more easily and hence has a lower learning capacity. In particular, we found that when $\gamma =20$, the loss function stops decreasing and becomes nearly zero when the prediction probability (confidence level) is greater than 0.2, which means that the model stops learning. Obviously, this is undesired. In practice, for any trainable models, we would expect the prediction confidence level to be higher than 50%. Because the output of softmax always is bounded in [0,1], if its value exceeds 0.5 and is indeed a correct prediction, then we can say that the model has learned the input data quite well. Accordingly, it is desired to see that the learning activity of the model continues (i.e., the loss decreases) relatively fast as long as the prediction probability is smaller than 0.5. However, when the prediction probability is greater than 0.5, the decrease amount in the loss function should be relatively slow. In light of this, $\gamma \in [{0,5})$ is recommended for training Lite3D. In addition, a rule-of-thumb for setting ${\alpha }_{i}$ is as follows: the smaller the amount of data in the *i*th class, the larger ${\alpha }_{i}$ is used, so the learning can be *focused* on classes with less data. This property is quantified by Eq. (9), where ${S}_{total}$ and ${S}_{i}$ denote the total number of training data and that in the *i*th class, respectively.

8 $$FL\left(y, A\left(x\right)\right)=-\sum_{i}{{\alpha }_{i}{\left(1-A({x}_{i})\right)}^{\gamma }y}_{i}{\log}A({x}_{i})$$

9 $${\alpha }_{i}=1-\frac{{S}_{i}}{{S}_{total}}$$

## Prediction phase

In this study, a multi-thread tracking algorithm is developed to work with YOLOv4_tiny to provide the sequences of ROIs for behavior recognition of multi-targets. A set ${\varvec{T}}=\left\{{t}_{j}\right\}$ is used to keep all the targets being tracked frame by frame. Initially, ${\varvec{T}}=\boldsymbol{\varnothing }$**.** Within each frame, objects (e.g. fish) ${\varvec{X}}=\left\{{x}_{i}^{d}\right\}$ detected by YOLOv4_tiny at *d*th frame are subjected to the tracking Algorithm (see Supplementary Algorithm_S2) whereby multiple targets are tracked for $k$ frames, ROIs of which will be subjected to the cut-paste-warp scheme, and the resulting sequence will be fed into the trained Lite3D for behavior recognition. Note that a prediction ROI is given for any tracking-loss frame. More details on the tracking algorithm are given as follows.

10 $$j=\left\{IoU\left({\varvec{T}}, {x}_{i}\right)>\alpha \right\}\cap \{Dist\left({\varvec{T}}, {x}_{i}\right)<\beta \}$$

Any object ${x}_{i}$ is to be matched with all threads in ${\varvec{T}}$ using Eq. (10), whereby elements ${t}_{j}$ having a sufficiently large IoU (intersection over Union) with ${x}_{i}$ are further subjected to a distance checking, i.e., the element in the thread ${\varvec{T}}$ that matches ${x}_{i}$ and has a distance from ${x}_{i}$ being smaller than the threshold $\beta$ (in pixels) is picked.

Note that the values of $\alpha$ and $\beta$ can be varied according to the species of the target, e.g. in dealing with speedy creatures, a smaller $\alpha$ allows more candidates to be selected for the distance checking, thus avoiding failure tracking. During the tracking, three working sets ***A*** (*Add*), ***U*** (*Update*), and ***P*** (*Predict*) are used to keep track of new, aborted threads, and the estimation for the tracking loss, respectively. If ${x}_{i}$ does not match any thread, then ${x}_{i}$ is a new target that needs to be added to ${\varvec{A}}$. By setting the prediction set ${\varvec{P}}$ as ${\varvec{T}}-{\varvec{U}}$, we can save computation time, because that way we only need to make predictions for the objects in ${\varvec{P}}$, rather than predicting for all detected objects as in other tracking methods. In all experiments presented in this paper, we simply use the linear predictor in both $x$ and $y$ directions as the strategy for predicting the next positions of tracking objects. Following the prediction, if there are newly detected objects, add them to ${\varvec{T}}$. Without loss of generality, if a target ${t}_{j}$ is lost for *r* successive frames, the tracking of ${t}_{j}$ can be aborted and removed from ***T***. In this study, $r=k/2.$ Also, a data structure of a first-in-first-out stack is assigned to each ${t}_{j}$ for keeping coordinates data of ROIs, and the top ten ROIs form a sequence $\overline{{\varvec{d}} }$ and are sent to Lite3D for recognition, as shown in Fig. 4.

It is interesting to visualize the effectiveness of Lite3D in extracting useful features for behavior recognition. Supplementary Fig. S9 illustrates the 3-D distribution map of the classification results, i.e., the best tree principal features derived by applying the Principal Component Analysis to the output features at the flatten layer in Supplementary Fig. S6. For comparison, we trained two separate models of Lite3d using entropy loss and focal loss, respectively. Clearly, the nonuniform density distribution in Supplementary Fig. S9 has justified that there exists a difference in importance among the features, and some features indeed carry more information than others. In addition, the more concentrated density distribution in Supplementary Fig. S9b than that in Supplementary Fig. S9a indicates that the focal loss outperforms the ordinary entropy loss.

### Supplementary Information

Supplementary Information.

## Data Availability

The raw dataset generated and/or analyzed during the present study is now available and can be accessed through the link: http://140.121.135.204/aicenter/publications.html, under the repository name of *Anomaly behavior recognition of underwater creatures using Lite3D full-convolution network*. Currently, the dataset includes seven behavior categories (as those shown in Supplementary Fig. S7) and will be continuously expanded in the future by the AI Research Center at NTOU. For dataset of creatures other than those presented in this article, readers are encouraged to visit https://ai-center.ntou.edu.tw/project/dataset.

## Code availability

For readers who are interested in acquiring the training set, test set as well as augmented image data used in this paper, the programs needed for preparing the above datasets are accessible through Github https://github.com/softcomputinglab520/Anomaly-Behavior-Recognition-of-underwater-creatures-Using-Lite-3D-Full-Convolution-Network.
