# Supplementary material for: Anomalous behavior recognition of underwater creatures using lite 3D full-convolution network
Source: Sci Rep. 2023 Nov 16;13:20051. doi: 10.1038/s41598-023-47128-2 (PMC10654714; doi:10.1038/s41598-023-47128-2)
Supplement: Supplementary file 1 — Supplementary Information. [file 41598_2023_47128_MOESM1_ESM.docx]

**Supplementary Materials**

| Supplementary Table S1 Profile of the private (labeled) dataset | | | | | | | |
| --- | --- | --- | --- | --- | --- | --- | --- |
|  | Cobia | | | Tilapia | | | |
| Category | normal | lifeless | anomalous | normal | grinding | cartwheeling | side_swim |
| Training | 1126 | 3543 | 36 | 4223 | 53 | 143 | 42 |
| Testing | 290 | 853 | 14 | 1075 | 13 | 37 | 10 |
| Subtotal | 1416 | 4396 | 50 | 5298 | 66 | 180 | 52 |
| Total | 5862 | | | 5596 | | | |

| **Supplementary Algorithm_S2** | | | |
| --- | --- | --- | --- |
| **Initialization:** $\boldsymbol{A}$, $\boldsymbol{U}$, $\boldsymbol{P}$**,** *k*=0 | | | |
| **Input:** A set of detected objects $\boldsymbol{X}=\{x_{i}\}$, *i*=*1, 2,..m* | | | |
| **Output:** Set of tracking targets $\boldsymbol{T}=\{t_{j}\}$ | | | |
| **do** | | | |
|  | $\boldsymbol{A}=\emptyset, \boldsymbol{U}=\emptyset, \boldsymbol{P}=\emptyset$ | | |
|  | *k* = *k*+*1* | | |
|  | **for** $i= 1 to m$ **do** | | |
|  |  | Get matched index $j$using Eq. ([10](#eq6)) | |
|  |  | **if** *m*$atch is successful$ | |
|  |  |  | $\boldsymbol{U}\leftarrow\boldsymbol{U}\cup\{t_{j}\}$ |
|  |  | **else** | |
|  |  |  | $\boldsymbol{A}\leftarrow\boldsymbol{A}\cup\{x_{i}^{d}\}$ |
|  |  | **endif** | |
|  |  | **if** $tracking of t_{j} is aborted$ | |
|  |  |  | $\boldsymbol{T}\boldsymbol{= T -}t_{j}$ |
|  |  | **endif** | |
|  |  | $\boldsymbol{P}=(\boldsymbol{T}-\boldsymbol{U})$ | |
|  |  | update$\boldsymbol{(T\cap U)}$ | |
|  |  | **if** $\boldsymbol{P\neq\emptyset}$ | |
|  |  |  | Predict ***P*** |
|  |  | **endif** | |
|  |  | update$\boldsymbol{(T}\boldsymbol{\cap}\boldsymbol{P)}$ | |
|  |  | $\boldsymbol{T}\leftarrow\boldsymbol{T}\cup\boldsymbol{A}$ | |
|  | **end** | | |
| **While** the prediction process is not terminated | | | |

**Supplementary Figure S3**. Posture definition. (a)Body parts mistakenly detected using DCG-DTW^14^. (b)Fish swimming toward the reader. (c) swimming away far from the reader.


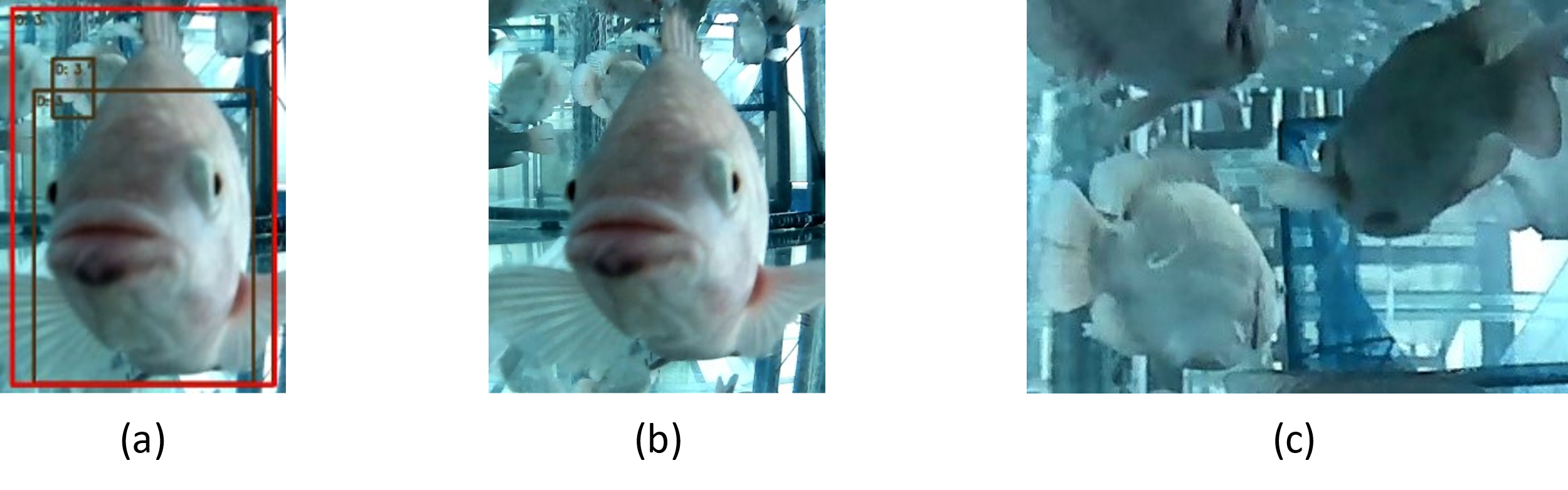

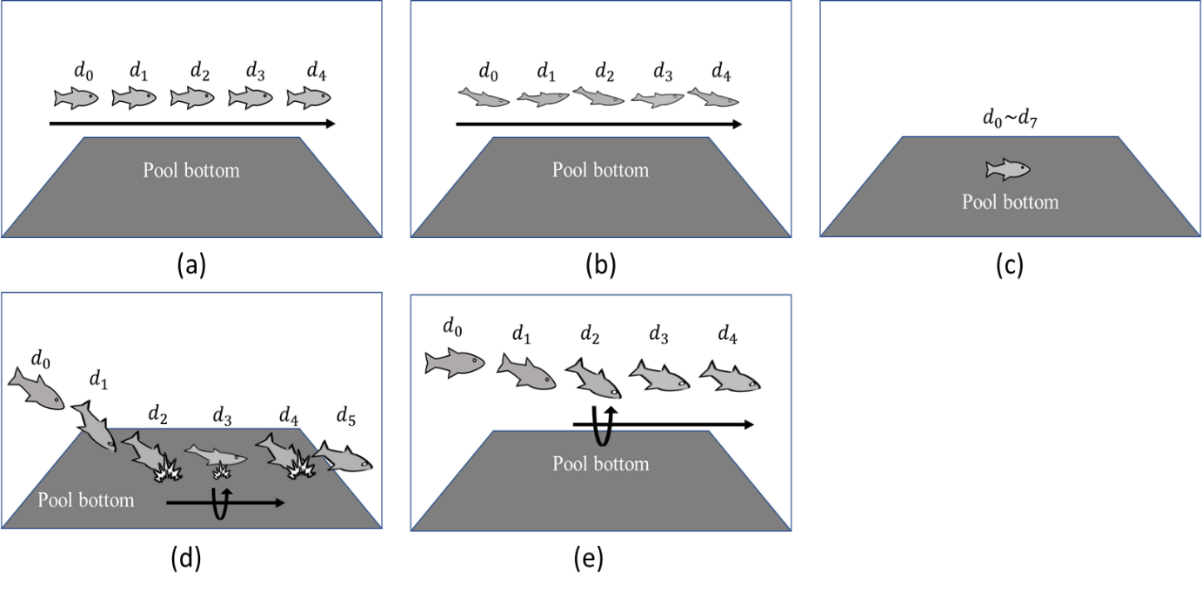


**Supplementary Figure S4**. Five categories (a) normal (b) side_swim (c) lifeless (unmoved at the pool bottom) (d) grinding the pool bottom (e) cartwheeling.


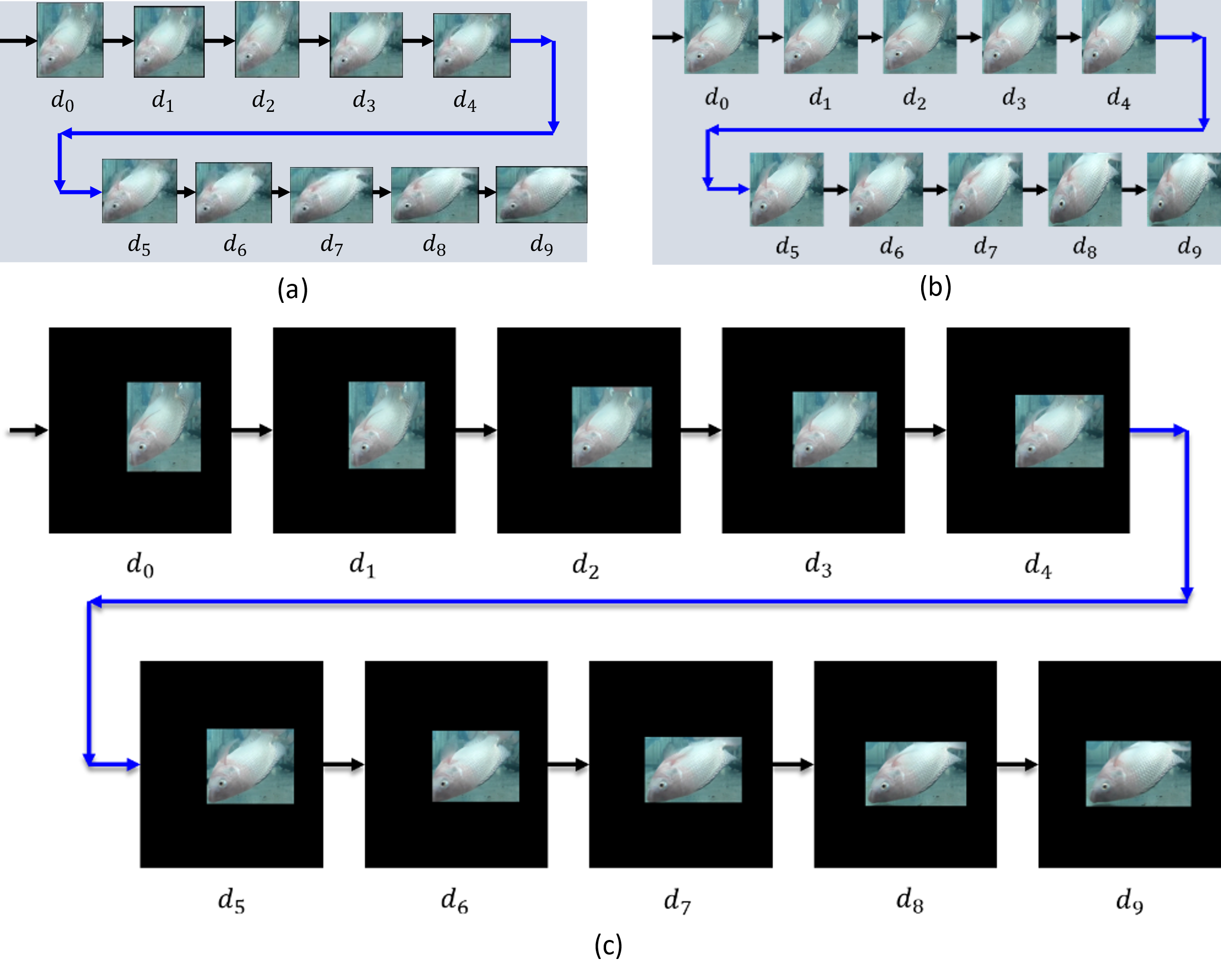


**Supplementary Figure S5**. A swimming sequence (a) originally cutout ROIs (b) warped ROIs that become equally in size. (c) Pasted ROIs of (a) with blacken background.


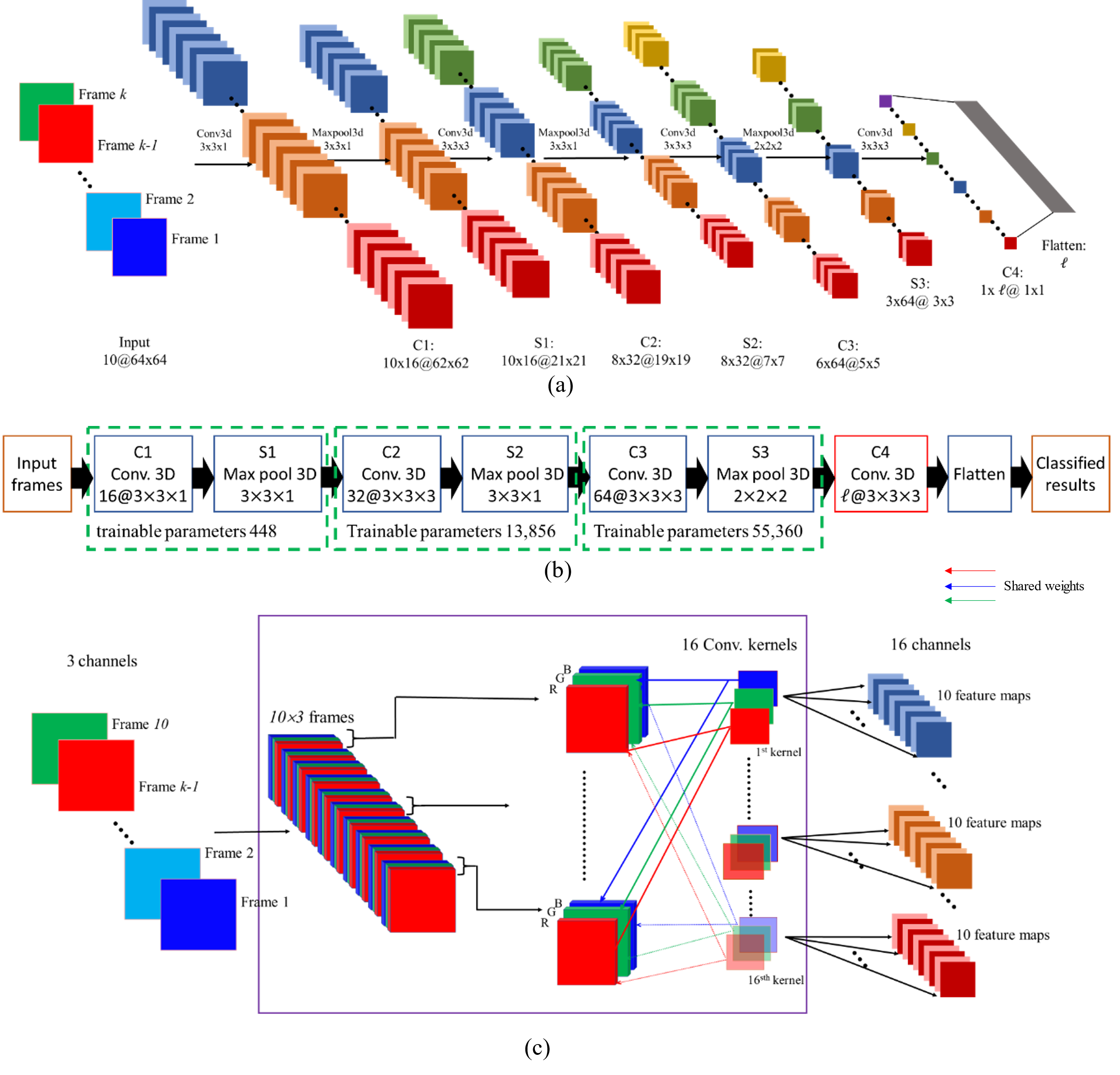


**Supplementary Figure S6**. Network architecture of Lite3D. (a) tensor size of input, feature maps, and output of Lite3D; (b) configuration of each layer in Lite3D;(c) 3×3×1 convolution for frames at $C_{1}$layer


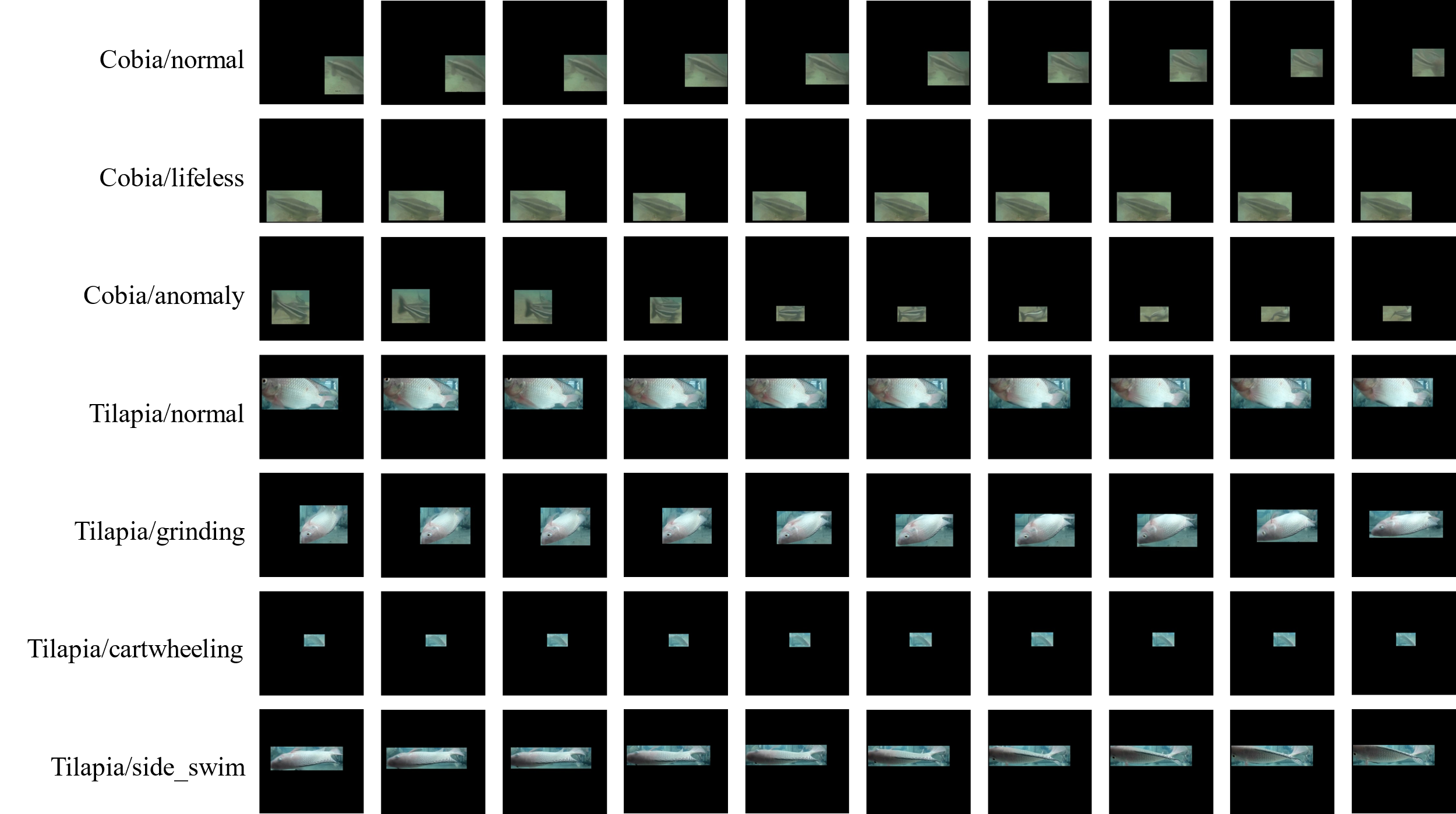


**Supplementary Figure S7**. Sequential data for the seven different categories of behaviors in our dataset


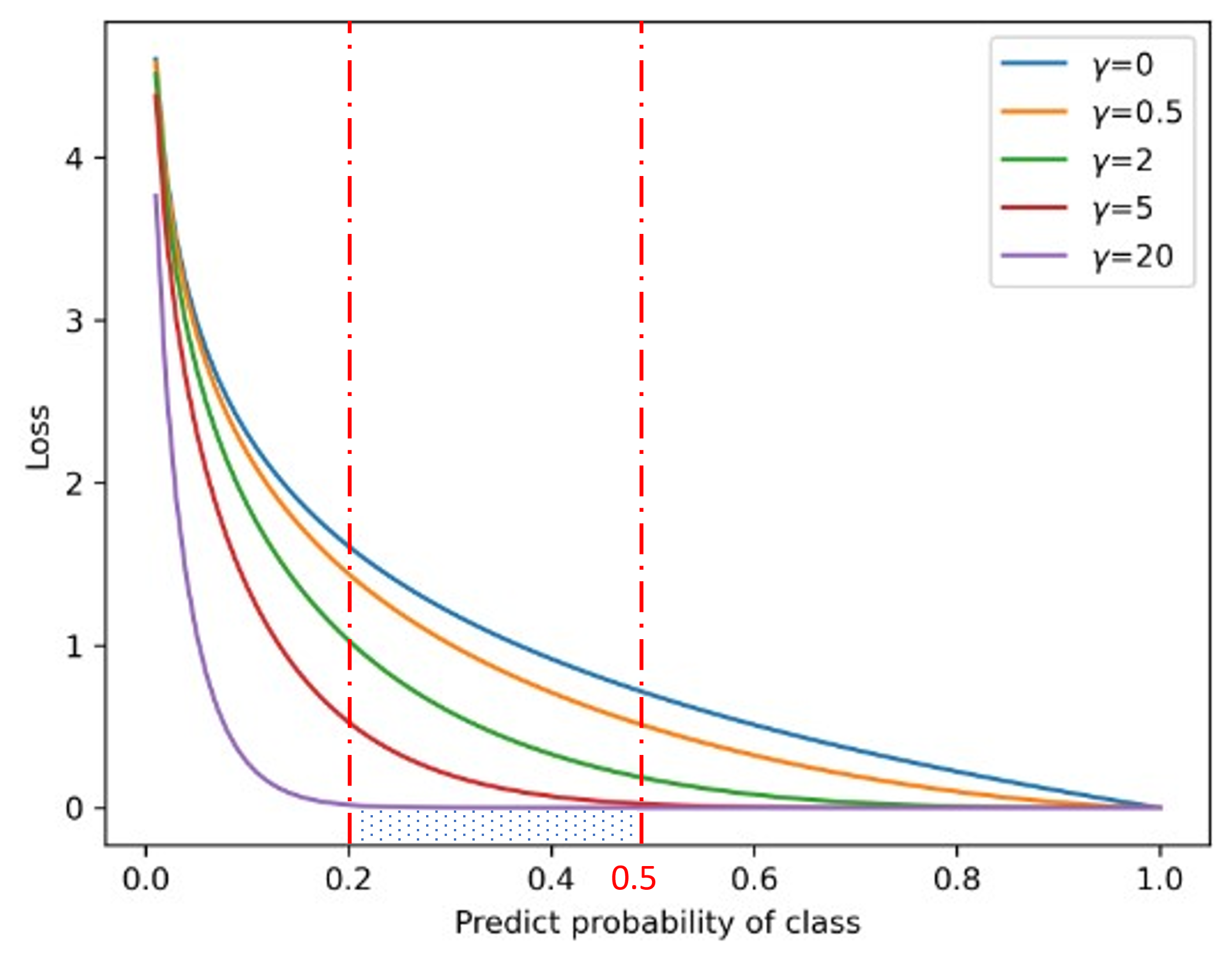


**Supplementary Figure** **S8**. In the case of $\alpha_{i}=1$, the influence of different $\gamma$ values on focal loss


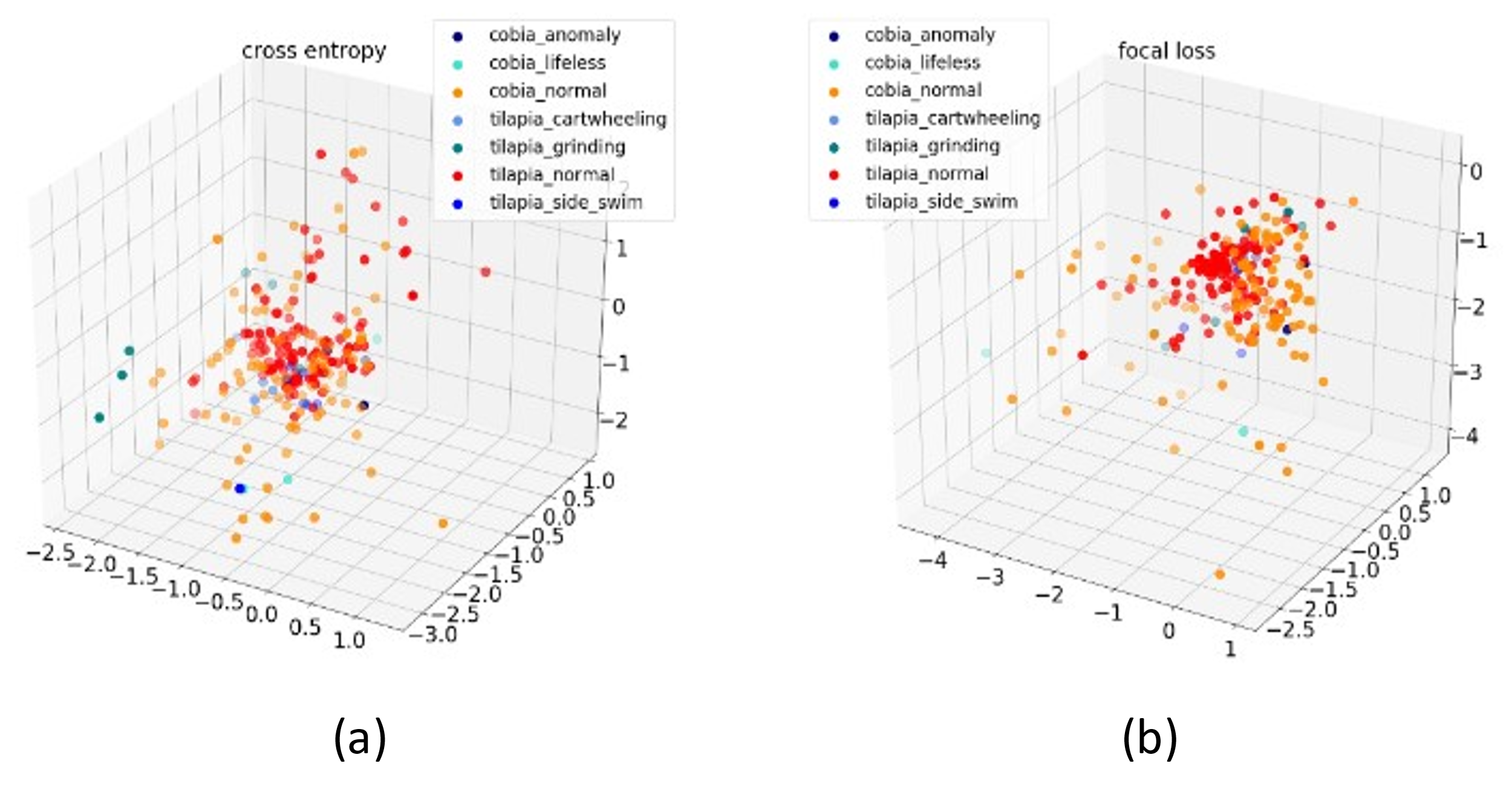


**Supplementary Figure S9**. Distribution map of test data in 3D feature space. (a) trained with cross entropy (b) trained with focal loss
